# Spatial Heterogeneity in Positional Errors: A Comparison of Two Residential Geocoding Efforts in the Agricultural Health Study

**DOI:** 10.3390/ijerph18041637

**Published:** 2021-02-09

**Authors:** Jared A. Fisher, Maya Spaur, Ian D. Buller, Abigail R. Flory, Laura E. Beane Freeman, Jonathan N. Hofmann, Michael Giangrande, Rena R. Jones, Mary H. Ward

**Affiliations:** 1 Division of Cancer Epidemiology and Genetics, National Cancer Institute, Bethesda, MD 20892, USA; mss2284@cumc.columbia.edu (M.S.); ian.buller@nih.gov (I.D.B.); freemala@mail.nih.gov (L.E.B.F.); hofmannjn@mail.nih.gov (J.N.H.); rena.jones@nih.gov (R.R.J.); wardm@exchange.nih.gov (M.H.W.); 2 Westat, 1600 Research Blvd., Rockville, MD 20850, USA; abigail.flory@nih.gov (A.R.F.); MichaelGiangrande@westat.com (M.G.)

**Keywords:** geocoding, positional error, rural location, spatial analysis, exposure assessment, environmental epidemiology

## Abstract

Geocoding is a powerful tool for environmental exposure assessments that rely on spatial databases. Geocoding processes, locators, and reference datasets have improved over time; however, improvements have not been well-characterized. Enrollment addresses for the Agricultural Health Study, a cohort of pesticide applicators and their spouses in Iowa (IA) and North Carolina (NC), were geocoded in 2012–2016 and then again in 2019. We calculated distances between geocodes in the two periods. For a subset, we computed positional errors using “gold standard” rooftop coordinates (IA; N = 3566) or Global Positioning Systems (GPS) (IA and NC; N = 1258) and compared errors between periods. We used linear regression to model the change in positional error between time periods (improvement) by rural status and population density, and we used spatial relative risk functions to identify areas with significant improvement. Median improvement between time periods in IA was 41 m (interquartile range, IQR: −2 to 168) and 9 m (IQR: −80 to 133) based on rooftop coordinates and GPS, respectively. Median improvement in NC was 42 m (IQR: −1 to 109 m) based on GPS. Positional error was greater in rural and low-density areas compared to in towns and more densely populated areas. Areas of significant improvement in accuracy were identified and mapped across both states. Our findings underscore the importance of evaluating determinants and spatial distributions of errors in geocodes used in environmental epidemiology studies.

## 1. Introduction

In environmental epidemiology studies of spatially- and temporally-referenced exposures, geocoding of residential addresses is typically used to assign latitude and longitude coordinates in a Geographic Information System (GIS) as a first step in an exposure assessment. However, relatively few studies have compared geocoding methods or assessed the accuracy of geocodes [1,2,3,4,5]. In rural areas, residential proximity to crop fields treated with pesticides can increase pesticide concentrations in homes, with implications for the risk of adverse health outcomes [6,7,8]. Positional errors and imprecision in geocoding may result in exposure misclassification, with implications for exposure assessment of drinking water sources [3,4], non-occupational agricultural pesticides [2,3,9], air pollution [5,10], and other environmental exposures [11,12].

Positional accuracy is defined as the distance between the residence’s location, best determined using a field survey method such as a Global Positioning System (GPS) device or digitally enhanced aerial orthoimagery [13,14,15], and the geocoded point location linked to the address [16]. Evaluating variability in the accuracy of geocoding methods allows investigators to evaluate the potential impact of this variability on their analyses of environmental exposures at the residence and human health [17,18,19]. The assessment of geocoding accuracy is particularly important in rural areas, where studies have shown greater positional error than in urban or suburban areas [20,21]. In addition to positional accuracy, geocoding quality is quantified by a match rate that is the proportion of addresses that can be geocoded [22]. Positional accuracy and match rates of rural addresses geocoded using street databases tend to be worse than urban or suburban addresses due to the larger street segments and distances between the house and public road, which may magnify interpolation errors [2]. Match rates also tend to be lower in rural areas compared to urban areas in part due to the prevalence of rural routes and Post Office (PO) boxes [16], which cannot be located accurately by geocoding [3,23].

The match rate and positional accuracy of geocoding depend on the assumptions behind the matching algorithms as well as the accuracy of the underlying reference (street) datasets [24,25]. The address is matched to a spatial coordinate in the street reference dataset through a matching process that enables matching at different levels of precision based on the address input information available, while a minimum match score that reflects the level of confidence in locating the address must be met or exceeded [26,27]. These algorithms and datasets have improved over time, highlighting the need to assess how older geocode coordinates correspond to newer geocode coordinates obtained with more accurate and complete street databases [25]. In this study, we compared coordinates obtained from an older geocoding effort (Geocode database Version 1: 2012–2016) to a newer geocoding effort (Geocode database Version 2: 2019) in the Agricultural Health Study (AHS), a large cohort of pesticide applicators and their spouses in Iowa (IA) and North Carolina (NC), USA. The primary objective of this study was to compare the geocodes to gold-standard coordinates (GPS and rooftop coordinates) in order to evaluate positional accuracy by geocode match status and rurality and determine if geocodes improved over time. We also conducted a spatial analysis to determine geographic areas with improvements in positional accuracy between the two geocoding efforts.

## 2. Materials and Methods

### 2.1. Study Population

The AHS is a prospective cohort of 52,394 licensed private pesticide applicators residing in Iowa and North Carolina, 32,345 of their spouses, and 4916 commercial applicators in Iowa. Details about the study design and cohort have been described [28]. Notably, over 80% of private pesticide applicators were enrolled in the study in Iowa and North Carolina, two states with large farming populations in the Midwest and Southeast, respectively [29]. To date, the AHS has included four phases, starting with enrollment (Phase 1) in 1993–1997 and followed by three follow-up interviews. At Phase 1, applicators and spouses provided the address of their current residence. Applicators and spouses also confirmed or provided an update to their address (including updating rural route addresses with street addresses) during follow-up interviews at Phase 2 (1999–2003), Phase 3 (2005–2010), and Phase 4 (2013–2015). Additionally, address changes were recorded during tracing efforts from 2012 to 2018. A street address from later follow-up surveys was substituted when an earlier address was a rural route or PO Box if the participant had not moved. As spouses resided at the same set of addresses as applicators at Phase 1, this analysis is limited to only private and commercial pesticide applicators (N = 36,792 in IA; N = 20,518 in NC).

### 2.2. Geocoding Process

Phase 1 enrollment address geocodes are being used to assess environmental exposures including nitrate in private wells and proximity to concentrated animal feeding operations for studies of cancer incidence [11,30,31]. The Phase 1 addresses have been geocoded several times and have been compiled into distinct geocode database versions. In Version 1, IA and NC addresses were batch- and interactively geocoded with the Esri ArcGIS Geocoding Engine software using the NAVTEQ 2011 street database in 2012 for IA and the HERE (formerly Navteq) 2015 street database in 2016 for NC [3]. During batch geocoding, an input table of participant addresses was automatically matched to the reference street database, primarily using the Point Address (not widely used until 2014) and Street Address locators. Point Address locators use a database of address points based on land parcel data; matches return latitude and longitude coordinates for an address, typically in the center of the land parcel for the property. Street Address locators use a spatially-referenced street database that provides the range of possible addresses for a street segment on the odd and even sides of the street. The input address number’s latitude and longitude coordinates are assigned by interpolation within the address range on the street segment. Street Address settings for batch-matched addresses included a street offset of 30 feet (9.1 m) from the street centerline and an end offset (squeeze factor) of 10% of the length of the street segment. The street centerline and end offsets are used to approximate the distance of the home away from the center of the street and to avoid addresses at the end of a street from being placed too close to an intersecting road, respectively [26]. The minimum match score/tolerance, a setting with a range of 0 to 100 to control how closely addresses match to their most likely candidate, was set to 80, the default value. Other match status types (Street Centroid, ZIP Centroid, City Centroid) were typically returned when only a partial address was available or if the street address did not exist within the range of existing address numbers on the street.

In the first geocoding effort, we conducted interactive geocoding when a street address was known but did not return a match with the Point Address or Street Address locators. Interactive geocoding is the manual process of correcting address information and assigning geographic coordinates. Addresses were searched using Google Maps^®^ and other internet sites to identify potential spelling errors, formatting inconsistencies, or areas where the reference data were missing or incomplete. If the address was in the form of a rural route or PO Box, interactive geocoding was not used because the location could not be improved beyond the ZIP code centroid.

IA and NC Phase 1 addresses were batch geocoded again in 2019 (database Version 2). Batch geocoding in 2019 used the Esri ArcGIS Geocoding Engine software and HERE StreetMap Premium 2018 V1, a street reference dataset in which each locator had its own default match score and offset. The Point Address locator was used for any address meeting the minimum match score of 93. The minimum match score used for the Street Address locator was 85. The Street Address locator used a default offset of 25 feet (7.6 m) from the center line and an end offset of 10%. The quality of match statuses for both geocoding efforts was considered good if the status was Point Address, Interactive (Version 1 only), or Street Address; other match statuses were considered poor.

### 2.3. Positional Accuracy: Rooftop Coordinates for a Subset of Iowa Participants

Rooftop locations of enrollment addresses for IA AHS participants who resided in 14 counties in western Iowa (Supplemental Figure S1) were assigned by researchers at the University of Iowa/Iowa Field Station by comparing geocoded participant addresses (geocoded with ArcGIS) to latitude/longitude data from the Iowa Roof-top Coordinates project. Orthoimagery, ESRI’s street database, Iowa E911 address data, and Iowa spatial parcel information were also used to aid in the assignments [3]. A total of 3566 applicators had rooftop coordinates linked to their geocoded address. Of these participants, 3467 and 3368 had a good match status for their Version 1 and Version 2 geocodes, respectively.

### 2.4. Positional Accuracy: Comparison to Home GPS Readings

The Biomarkers of Exposure and Effect in Agriculture (BEEA) study is a molecular epidemiologic sub-study in the AHS (N = 1681), in which most participants had a GPS reading taken at the front entrance of their home at the time of an in-home interview [32]. Residence locations were primarily in eastern IA but covered most of the state in NC (Supplemental Figure S1). Quality assurance and quality control were performed on the GPS coordinates for participants if the geocode and GPS locations were ≥500 m apart (n = 304). GPS readings were checked against the street addresses to ensure they were located on the correct street and city. If address attributes did not match, the GPS locations were not considered a gold standard for computing positional error. After excluding participants who moved since enrollment and those without GPS coordinates or verified coordinates at the Phase 1 address, 1258 participants (991 in IA, 267 in NC) were eligible for inclusion in this analysis. Of these participants, 1234 and 1192 had a good match status for their Version 1 and Version 2 geocodes, respectively.

### 2.5. Statistical Analysis

For participants with a good geocode match status in both geocoding efforts (N = 32,035 or 87.1% in IA, N = 16,919 or 82.5% in NC), we calculated distances between Version 1 and Version 2 geocodes in ArcGIS. Separately for Version 1 and 2 geocodes with a good match status, we calculated positional error (Z), defined as the Euclidean distance between the geocode and one of the three gold-standard coordinate datasets (GPS readings in IA and NC and rooftop coordinates in IA). Means and distributions of these distances and positional errors were calculated by the type of good geocode match status. Improvement was assessed as the difference in positional error between Version 1 and Version 2 geocodes if both were a good match status (calculated as Z_Version1_-Z_Version2_). Linear regression models were used to evaluate whether rural status and population density were predictive of the positional error of Version 2 geocodes and improvement in positional error between geocoding efforts. For modeling, positional error improvement was parameterized as a natural logarithm-transformed ratio (ln(Z_Version2_/Z_Version1_)). Rural status was determined based on whether the GPS or rooftop coordinates were located within a Census 2000 Incorporated Place (considered non-rural) or located outside an Incorporated Place (rural). Block-level population density at the GPS or rooftop coordinates was obtained from the 2010 Census. Moran’s *I* statistic was used to examine the spatial autocorrelation of residuals from the linear regression models (Euclidean inverse-distance weighting in meters with *p*-values based on a normal approximation).

### 2.6. Spatial Analysis

Full details of the spatial analyses are presented in Supplemental Appendix A. For linear regression models with significant spatial autocorrelation of the residuals, a simultaneous autoregressive error model was used with an adjacency matrix based on *k*-nearest neighbors to determine the coefficients and *p*-values for the two predictor variables. To provide a visual representation of areas with low and high positional error without showing participant locations (which are protected identifiable information), positional errors of the geocodes were spatially interpolated through kriging. The best-fitting semivariogram of natural log-transformed positional error between each gold standard and geocoding effort was selected using the gstat package in R (Ver 3.5.3). The improvement in positional error was evaluated spatially using a spatial relative risk function. We extended the conventional approach of the spatial relative risk function to compare a binary temporal grouping (Version 2 geocodes vs. Version 1 geocodes). We incorporated spatial densities that were weighted by the positional error (in meters); the “risk” is characterizing the spatial distribution of positional error in the Version 2 geocodes relative to Version 1 geocodes across a study area. To accomplish this, the spatial density of study participants for each of the three gold-standard coordinate sets was weighted by their positional error (Z) for both Version 1 and Version 2 geocodes. Next, the spatial relative risk function of the weighted spatial densities was calculated using the spatstat package in R (Ver 1.64-1). We present the natural log-transformed relative risk estimate (ln(E)) and its standard error (σ_ln(E)_), which we approximate using the delta method (σ_ln(E)_ = σ_E_/E) where the standard error of the relative risk estimate (σ_E_) is divided by the relative risk estimate (E) at each smoothed grid location in the study area. Significance in positional error improvement or deterioration, compared to an expectation of homogeneous relative risk (null value of E = 1), was determined as smoothed grid cells with a relative risk estimate (E) that exceeded a two-tailed 95% confidence interval under a normal approximation for the spatial relative risk function. This approach is based on similar assumptions by Hazelton and Davies (2009) but implemented for weighted spatial densities [33].

## 3. Results

### 3.1. Geocoding Process

Match status and match quality for both geocoding efforts are presented in Table 1, separately by state. Over 85% of addresses had a good geocode status with a higher percentage in Version 1 (IA = 91.5%, NC = 85.4%) compared to Version 2 (IA = 88.7%, NC = 82.5%) due to the interactive geocoding of Version 1 street addresses that did not have a good match status after batch geocoding. Though the Point Address locator was used minimally in the Version 1 effort, it was prioritized in batch geocoding processes by 2019, and thus most addresses were geocoded to this match type in 2019 (IA = 72.1%, NC = 68.8%).

Distances between Version 1 and Version 2 geocoded coordinates with good match statuses varied by state (Table 2). Overall, the median distance between geocode pairs was 170 m (interquartile range, IQR: 53–293 m) for IA and 87 m (IQR: 39–202 m) for NC. Median distance between geocodes was generally lowest when match statuses were the same, such as when both were Point Address (145 m in IA and 57 m in NC) or Street Address (2 m in IA and 0 m in NC). Though 95% of the distances between pairs were under 936 m for IA and 556 m for NC, there were several extreme values (maximums of 48.5 km in IA and 35.6 km in NC).

### 3.2. Positional Accuracy

Positional error calculations for Iowa participants with rooftop coordinates are presented in Table 3 for both geocoding efforts. Ninety-seven percent had a good match status in the Version 1 (mostly Street Address) and Version 2 (mostly Point Address) geocoding efforts. Overall, the median positional error was 124 m (IQR: 60–290 m) for the Version 1 geocodes and 65 m (IQR: 30–171 m) for the Version 2 geocodes. For Version 2, Point Address geocodes were more accurate than Street Address geocodes based on each percentile cutpoint (5, 25, 50, 75, 95%). For participants with good quality geocodes in both efforts, 67.4% of geocodes had less positional error in the Version 2 geocoding effort; median improvement between the geocoding efforts was 41 m (IQR: −2 to 168 m).

Positional errors computed from the GPS readings are presented in Table 4. Overall, geocodes were more accurate in the Version 2 geocoding effort than the Version 1 effort as quantified by the mean, median, and other percentile distributions of positional error. Geocodes were also more accurate for NC compared to IA for both Version 1 and Version 2 datasets. Additionally, in the Version 2 dataset, the median positional errors of geocodes assessed with the Point Address locator were more accurate than Street Address geocodes for both IA (Point Address = 166 m; Street Address = 193 m) and NC (Point Address = 42 m, Street Address = 178 m). For IA participants, the median improvement between efforts was 9 m (IQR: −80 to 133 m), and positional error decreased for 53.2% of the participants. For NC participants, the overall median improvement was 42 m (IQR: −1 to 109 m), and 71.4% had a more accurate Version 2 geocode.

Supplemental Tables S1 and S2 show the positional error of geocodes by rural status in both IA and NC as assessed by comparisons to rooftop or GPS coordinates, respectively. Across both states, both geocoding efforts and both reference coordinate sets (rooftop or GPS) geocodes for non-rural addresses were more accurate than geocodes for addresses in rural areas. For example, the median positional error for rural addresses geocoded in the Version 2 dataset in IA was 173 m (IQR: 73–242 m), but it was 25 m (IQR: 14–59 m) for the non-rural addresses geocoded in the same effort. Though the positional error of geocodes was comparable between non-rural addresses in IA and NC, the accuracy of rural geocodes was much higher in NC (Median = 48 m; IQR: 25–158 m) than in IA (Median = 173 m; IQR: 73–242 m) in the Version 2 dataset. Improvement in geocoding accuracy between the 1st and 2nd geocoding efforts was seen in both the rural and non-rural geocodes in both states.

Using linear regression models, we found that rural status and population density were significant predictors of positional error in the Version 2 geocodes and of improvement in positional error between geocoding efforts in IA (Table 5). For the IA rooftop coordinates, increased population density resulted in smaller positional errors (est = −4.78 m per 100 persons/km^2^ change; *p* = 0.05) as well as improvement in positional error between the two geocoding efforts (est = 0.98; *p* < 0.01). For the IA GPS comparisons, non-rural location was predictive of lower positional error (estimate = −507.8 m; *p* = 0.04) and improvement (improvement ratio = 0.69; *p* = 0.06). For the GPS comparisons in NC, a state with a higher overall population density, non-rural location and increased population density decreased positional error; however, neither variable was significant. Significant spatial autocorrelation of residuals was observed in the improvement models using the IA rooftop coordinates (Moran’s *I* = 0.076; *p* < 0.01) and IA GPS coordinates (Moran’s *I* = 0.012; *p* < 0.01); plots of the global Moran’s I are presented in Supplemental Figure S2.

**Figure 1:**
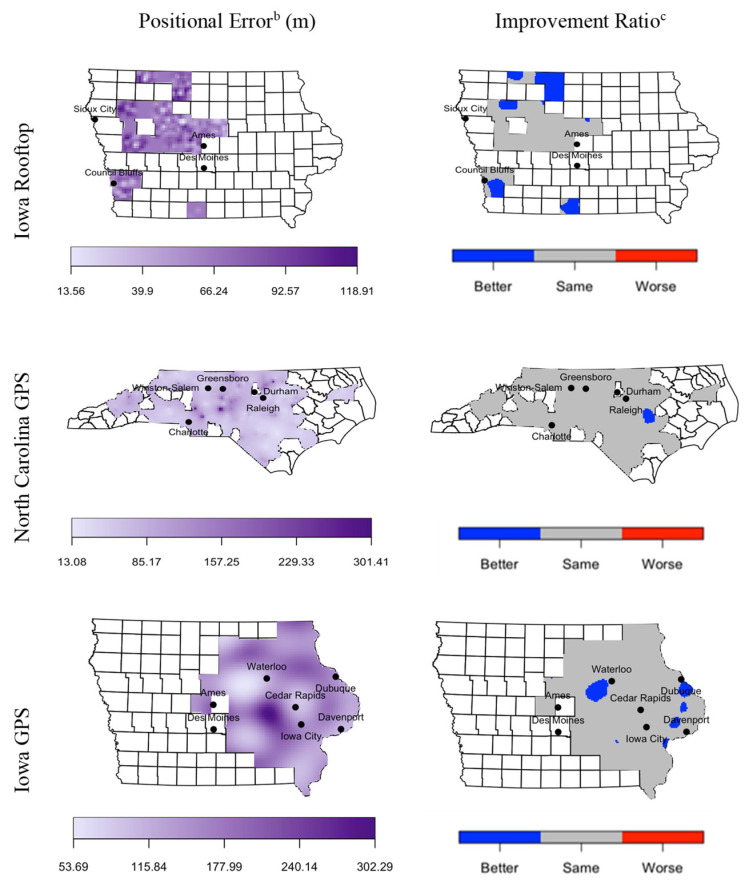
Spatial distribution of positional error of Version 2 geocodes and positional error improvement between Version 1 and Version 2 geocodes^a^ for Iowa rooftop coordinates and Iowa and North Carolina GPS coordinates. ^a^ Version 1: enrollment addresses were geocoded in 2012 for Iowa and in 2016 for North Carolina. Version 2: addresses were geocoded in 2019 for both states. ^b^ Figure denotes the spatially kriged positional error of Version 2 geocodes. Darker colors denote higher values of positional error. ^c^ Figure denotes significant improvement ratio, which is calculated as the estimated spatial relative risk of Version 2 geocode and Version 1 geocode (Z_Version2_/Z_Version1_; weighted by their respective positional error) that exceeds an asymptotic normal null expectation of homogenous relative risk. Blue-colored areas denote significant improvement in positional error between Version 1 and Version 2 geocodes, grey-colored areas denote insignificant change, and red-colored areas denote significant deterioration (which was not observed).

Spatially smoothed maps of positional error and improvement for each of the three reference sets are provided in Figure 1. No clear area-wide patterns emerged from the positional error maps. We noted a significant improvement in positional error in several clusters in Iowa using the GPS reference set, with the largest cluster located in central Iowa, west of the city of Waterloo. There was also a significant improvement in positional error in one area of eastern NC located southeast of the city of Raleigh. Based on Iowa rooftop coordinates, there were multiple areas of significant improvement in positional error, including the entirety of Kossuth County in north-central Iowa.

## 4. Discussion

In this comparison of two geocoding efforts in the AHS, we found that the positional accuracy of the geocoded residential addresses improved in both IA and NC. We noted smaller positional errors for addresses in NC compared to Iowa, and we found greater positional error in rural and less densely populated areas. We found that positional errors were spatially autocorrelated in IA and that certain regions of both IA and NC had significantly improved geolocations between the two geocoding efforts.

Our findings show that the newer geocoding locators and street reference datasets can more accurately capture the location of residences, which is important for exposure classification in environmental epidemiology studies. Using gold-standard locations as assessed by rooftop coordinates or on-site GPS readings, batch-matched geocodes showed substantial improvement in the second geocoding effort in 2019 compared with the first effort in 2012 and 2016, for IA and NC, respectively. This improvement is partly explained by the shift from the predominant use of the Street Address locator for the Version 1 geocodes (86.5% for IA, 68.7% for NC) to the more accurate Point Address locator for Version 2 (72.1% for IA, 68.8% for NC). However, even addresses matched by the Street Address locator in 2019 showed an improvement in accuracy. Accuracy may have improved over time for several reasons, including data corrections or other updates, such as a change in address ranges for a street segment.

Use of the Point Address locator has increased the positional accuracy of geocodes, likely due to the inclusion of parcel data (property boundaries). When digital parcel data are used for geocoding, coordinates are assigned to an address based on the parcel centroid or the location of the major structure on the property [34]. However, our results may illustrate limitations of geocoding using parcel data in rural areas. The large positional errors for some Point Address geocodes in this study may have occurred due to the poor geocoding accuracy for addresses located in large parcels, which are common in rural areas [16,24,35].

The positional errors we observed for both time periods are comparable to geocoding errors observed in other studies that evaluated geocoded addresses in Iowa [2,36,37] and other US states and countries [16]. In a case-control study of non-Hodgkin lymphoma in Iowa, Ward et al. compared two geocoding methods to GPS measurements at homes and found similar positional errors (medians = 61 and 62 m) to those in our study [2]. Zimmerman et al. modeled the probability distribution of positional errors for 2354 rural addresses in Carroll County IA and found batch-geocoding procedures to have a median error of 168 m [36]. Ganguly et al. found median positional errors of 26 m for batch geocoding using both Bing Maps and ArcGIS for 160 homes exposed to traffic-related air pollution in Detroit, Michigan [35]. Comparing ArcGIS geocodes to residential coordinates obtained using aerial orthoimagery for 506 urban addresses in Saint Louis, Missouri, Schootman et al. found a median positional error of 31 m [13].

We observed greater positional error for rural addresses than for non-rural addresses (located within US Census incorporated places) in both IA and NC. Median positional error based on GPS for rural addresses in IA was more than three times that for non-rural addresses (173 m vs. 23 m), whereas rural and non-rural median errors were more similar in NC (48 m vs. 29 m). The positional error distributions by rural status that we observed were similar to a previous comparison of AHS geocoded addresses that were not limited to enrollment geocodes [3] and similar to the differences observed by Ward et al. for residences located in incorporated places (medians = 50 to 56 m) and rural areas (medians = 88 to 212 m). Others have also examined predictors that might explain the increased positional error observed in rural areas in Iowa and found that street intersection density was predictive of positional error [37]. Our regression models suggest that rural status and population density were predictive of improvement in geocoding accuracy between the two efforts in Iowa. Commercially available geocoding databases likely target improvements to areas with higher population density, which might partly explain the finding of lower positional error among rural NC addresses compared to those in IA. The higher population density in NC may also explain the lack of associations between rural status and population density with positional accuracy and improvement in our linear regression models.

Inaccurate geocodes contribute to exposure misclassification in epidemiological studies [2,3,4]. In an analysis within AHS, Jones et al. found that positional errors comparable to those in our evaluation reduced the sensitivity and specificity of environmental exposure estimates (proximity to row crops and animal feeding operations) even at large buffer sizes (5 km). In several exposure scenarios, they noted up to a 50% reduction in the odds ratio for a hypothetical nested case-control study when address-matched geocodes were used instead of gold-standard coordinates [3]. An analysis of proximity to corn and soybean crops in Iowa by Ward et al. using home GPS coordinates as gold-standard locations found that misclassification was greatest for distances of 100 m compared to 250 and 500 m [2]. An analysis of exposure to concentrated animal feeding operations in Carroll County, Iowa by Mazumdar et al. found median positional errors of 211 m and 46 m [12] that resulted in modest attenuation of a true odds ratio from 1.21 to 1.18 and 1.17 depending on the geocoding method [12]. In an investigation of geocoding methods in rural areas, Vieira et al. evaluated addresses that were erroneously geocoded to street segments not receiving public water supplies or to street segments serviced by a different public water system. Geocoding errors were primarily due to incorrect street numbers and did not result in much exposure misclassification as the entire lengths of the streets were serviced by the same public water system [4]. However, for areas serviced by small public water supplies such as the rural water cooperatives in IA and NC, connections to the public supply can vary from address to address on the same street. Thus, small positional errors could result in the assignment of the wrong drinking water source.

After accounting for population density and rural status, we found significant spatial autocorrelation of positional error improvement in IA. Although clear patterns did not emerge when mapping positional error in IA and NC, our findings of significant spatial autocorrelation in IA and a high degree of spatial heterogeneity are important observations, especially as this could result in spatial clustering of geocoding-related misclassification in future observational studies. Though we did not find significant spatial autocorrelation among models of positional error of Version 2 geocodes, other studies have shown spatial autocorrelation among positional errors from automated geocoding in Iowa [38] and Florida [24]. The clustering of improvements in positional error in certain areas in IA and NC suggests that improvements in geocoding processes or reference databases maintained by commercial geocoding firms are not uniform across large geographic areas.

The strengths of this study include the availability of gold-standard GPS and rooftop coordinates across the study area of both states and the use of both spatial and aspatial models to visualize and determine significant predictors of geocoding accuracy and positional error improvement. Though gold-standard coordinates were not available for all AHS participants, our large sample size of reference coordinates allowed for an examination of accuracy by state, rurality, and match status. The different years of geocodes for IA (2012) and NC (2016) in the Version 1 dataset limit direct comparisons of improvements over time between the states. We tested for spatial autocorrelation of model residuals to determine if another spatial predictor (i.e., beyond rurality and population density) was contributing to positional error and/or improvement. For the two models that showed spatial autocorrelation in the residuals, we performed simultaneous autoregressive error models in order to obtain unbiased coefficients and *p*-values for the effect of population density and rurality on geocode improvement. Though we were not able to provide maps with local Moran’s I because of our need to protect the confidentiality of participants’ locations, we visualized geocode improvement with spatial relative risk functions and provided global Moran’s I plots in the supplemental to further examine the spatial autocorrelation of the residuals. Our interpolated surface maps provide an overview of positional error across the study area; however, these maps should not be used to extract exact values. It should also be noted that the GPS receivers we used to verify geocodes have positional error that can range from 10 to 20 m [22]. Finally, this study was based in IA and NC in largely rural populations, and all of our results may not be generalizable to other US states. However, our findings of improvements in positional accuracy over time are likely to be relevant to studies in other locations.

## 5. Conclusions

Recent improvements in the spatial datasets and technology underlying geocoding processes have reduced the positional error in batch geocoded addresses, as demonstrated for AHS participants. Our results indicate that researchers maintaining long-running prospective cohorts may want to update participant geocodes as batch geocoding methods improve over time. Additionally, if available, rooftop coordinates and GPS readings are the most accurate locational data for residential addresses and should be utilized when available. These findings demonstrate the utility and importance of assessing the positional accuracy of geocoding methods (past and present) that are often used in exposure assessment for environmental epidemiology studies.

## Figures and Tables

**Table 1:** Geocode match status (number [N], percentage [%] for enrollment addresses, Agricultural Health Study applicators in Iowa (n = 36,792) and North Carolina (n = 20,518)).

	Version 1 ^a^			Version 2 ^a^		
	Match Status	N	%	Match Status	N	%
**Iowa**	Good Match Status					
	Interactive	1730	4.7	---	---	---
	Point Address	120	0.3	Point Address	26,528	72.1
	Street Address	31,811	86.5	Street Address	6119	16.6
	Total	33,661	91.5	Total	32,647	88.7
	Poor Match Status					
	Street Centroid	828	2.3	Street Centroid	895	2.4
	ZIP Centroid	2269	6.2	ZIP Centroid	3216	8.7
	City Centroid	24	0.1	City Centroid	24	0.1
	Total	3121	8.5	Total	4135	11.2
	Unassigned ^b^	10	0.0	Unassigned ^b^	10	0.0
	Overall	36,792		Overall	36,792	
**North Carolina**	Good Match Status					
	Interactive	281	1.4	---	---	---
	Point Address	3139	15.3	Point Address	14,112	68.8
	Street Address	14,100	68.7	Street Address	2815	13.7
	Total	17,520	85.4	Total	16,927	82.5
	Poor Match Status					
	Street Centroid	53	0.3	Street Centroid	220	1.1
	ZIP Centroid	1804	8.8	ZIP Centroid	2230	10.9
	City Centroid	1	0.0	City Centroid	1	0.0
	Total	1858	9.1	Total	2451	11.9
	Unassigned ^b^	1140	5.6	Unassigned ^b^	1140	5.6
	Overall	20,518		Overall	20,518	

^a^ Version 1: enrollment addresses were geocoded in 2012 for Iowa and in 2016 for North Carolina. Version 2: addresses were geocoded in 2019 for both states. ^b^ No coordinates were assigned during the geocoding process due to no match to any locator.

**Table 2:** Distance between Version 1 and Version 2 geocodes for addresses in Iowa and North Carolina by match status.

Version 1 ^a^ Match Status	Version 2 ^a^ Match Status			Distance (m) between Geocodes				
		N	Mean (SD)	Min	5%	Median (IQR)	95%	Max
**Iowa**								
Interactive	Point Address	914	498 (1839)	1	14	186 (55–355)	1170	25,987
	Street Address	279	1210 (2258)	6	21	284 (83–1129)	6348	16,728
Point Address	Point Address	105	157 (112)	2	24	145 (60–208)	382	579
	Street Address	8	170 (217)	6	6	74 (51–234)	640	640
Street Address	Point Address	25,081	389 (1214)	0	26	202 (88–326)	933	48,535
	Street Address	5648	241 (1658)	0	1	2 (2–45)	737	47,713
	Total	32,035	372 (1338)	0	2	170 (53–293)	936	48,535
**North Carolina**								
Interactive	Point Address	80	307 (892)	0	4	75 (19–173)	1470	7318
	Street Address	20	3530 (5394)	6	17	153 (46–9038)	14,202	15,237
Point Address	Point Address	2922	112 (137)	0	18	57 (36–130)	379	1451
	Street Address	12	567 (1652)	5	5	106 (14–192)	5809	5809
Street Address	Point Address	11,105	255 (865)	3	30	121 (63–243)	600	35,568
	Street Address	2780	196 (1228)	0	0	0 (0–23)	532	22,202
	Total	16,919	225 (892)	0	0	87 (39–202)	556	35,568

^a^ Version 1: enrollment addresses were geocoded in 2012 for Iowa and in 2016 for North Carolina. Version 2: addresses were geocoded in 2019 for both states.

**Table 3:** Positional error (m) of and improvement between Version 1 and Version 2 geocodes compared to rooftop coordinates for Iowa participants by match status.

Rooftop Coordinate vs. Geocode			Positional Error (m)				
	N	Mean (SD)	Min	5%	Median (IQR)	95%	Max
Version 1 Geocodes ^a^							
Interactive	130	315 (464)	4	23	162 (63–338)	1176	3809
Street Address	3337	386 (1074)	3	26	123 (60–287)	1227	15,172
Total	3467	383 (1058)	3	26	124 (60–290)	1218	15,172
Version 2 Geocodes ^a^							
Point Address	3038	102 (153)	0	6	64 (29–171)	247	6847
Street Address	402	417 (1506)	9	19	67 (37–176)	1266	14,796
Total	3440	139 (543)	0	6	65 (30–171)	265	14,796
Improvement ^b^	3368	245 (1021)	−14,317	−147	41 (−2–168)	1077	14,887

^a^ Version 1: enrollment addresses were geocoded in 2012 for Iowa and in 2016 for North Carolina. Version 2: addresses were geocoded in 2019 for both states. ^b^ Improvement calculated as the difference between the positional error of the Version 1 and Version 2 geocodes and limited to those with geocodes of good match status in both efforts.

**Table 4:** Positional error (m) of Version 1 and Version 2 geocodes compared to Global Positioning System (GPS) coordinates for Iowa and North Carolina participants by geocode match status.

GPS vs. Geocode				Positional Error (m)				
		N	Mean (SD)	Min	5%	Median (IQR)	95%	Max
**Iowa**	Version 1 Geocodes ^a^							
	Interactive	43	249 (242)	12	22	192 (79–325)	703	1174
	Point Address	3	138 (35)	99	99	147 (99–168)	168	168
	Street Address	922	400 (1154)	5	30	149 (77–329)	1012	15,609
	Total	968	392 (1128)	5	30	150 (78–328)	998	15,609
	Version 2 Geocodes ^a^							
	Point Address	866	199 (546)	3	17	166 (59–227)	467	15,567
	Street Address	82	720 (1734)	10	27	193 (76–451)	3376	11,884
	Total	948	244 (742)	3	17	167 (62–236)	508	15,567
	Improvement ^b^	934	148 (905)	−1765	−302	9 (−80–133)	615	12,102
**North Carolina**	Version 1 Geocodes ^a^							
	Interactive	1	92	-	-	-	-	-
	Point Address	43	97 (104)	10	24	61 (37–102)	320	550
	Street Address	222	330 (1458)	7	32	131 (81–252)	682	19,324
	Total	266	291 (1335)	7	27	117 (67–225)	616	19,324
	Version 2 Geocodes ^a^							
	Point Address	227	107 (166)	3	10	42 (23–122)	481	1147
	Street Address	31	838 (3451)	24	27	178 (71–325)	931	19,404
	Total	258	195 (1213)	3	11	46 (25–150)	515	19,404
	Improvement ^b^	258	61 (224)	−1002	−205	42 (−1–109)	414	1316

^a^ Version 1: enrollment addresses were geocoded in 2012 for Iowa and in 2016 for North Carolina. Version 2: addresses were geocoded in 2019 for both states. ^b^ Improvement calculated as the difference between the positional error of the Version 1 and Version 2 geocodes and limited to those with geocodes of good match status in both efforts.

**Table 5:** Regression models predicting positional error of Version 2 geocodes and the improvement ratio in positional error between Version 1 and Version 2 geocodes.

	Positional Error of Version 2 Geocodes		Improvement Ratio ^a^	
Model	Estimate	*p*-Value	Estimate	*p*-Value
**Iowa Rooftop**				
Intercept	158.1	<0.001	0.448	<0.001
Non-rural location ^b^	−53.1	0.131	0.892	0.246
Pop. Density (100 persons/km^2^) ^c^	−4.78	0.048	0.986	0.025
Moran’s I of residuals	0.001	0.409		
**Iowa GPS**				
Intercept	564.1	<0.001	0.856	0.006
Non-rural location ^b^	−507.8	0.037	0.708	0.079
Pop. Density (100 persons/km^2^) ^c^	6.36	0.710	1.011	0.445
Moran’s I of residuals	0.004	0.140		
**North Carolina GPS**				
Intercept	375.2	<0.001	0.504	<0.001
Non-rural location ^b^	−281.6	0.606	0.955	0.906
Pop. Density (100 persons/km^2^) ^c^	−28.1	0.596	1.022	0.572
Moran’s I of residuals	−0.003	0.944	0.019	0.141

^a^ Improvement ratio is calculated as the positional error of the Version 2 geocode divided by the positional error of the Version 1 geocode (Z_Version2_/Z_Version1_). A simultaneous autoregressive error model was used for Iowa Rooftop and Iowa GPS improvement models, as aspatial linear models showed significant spatial autocorrelation of the residuals based on the Moran’s I statistic (*p* < 0.001). ^b^ Non-rural location defined as the location being within a Census 2000 Incorporated Place. Estimate represents the change in positional error associated with a residence in a non-rural location. ^c^ Population density at the block level from the 2010 Census. Estimate represents the change in positional error associated with an increase in population density of 100 persons per km^2^ at the residence.

## Data Availability

Requests for data, including the data used in this manuscript, are welcome as described on the Study Website (https://www.aghealth.nih.gov/collaboration/process.html). Data requests may be made directly at www.aghealthstars.com; registration is required. The Agricultural Health Study is an ongoing prospective study. The data sharing policy was developed to protect the privacy of study participants.

## Supplementary Materials

The following are available online at https://www.mdpi.com/1660-4601/18/4/1637/s1, Figure S1: Number of AHS participants per county with gold-standard rooftop and GPS coordinates in Iowa and North Carolina, Figure S2: Global Moran’s I plots of residuals from Iowa GPS and Iowa Rooftop linear regression improvement ratio models, Table S1: Positional error (m) of Version 1 and Version 2 geocodes compared to rooftop coordinates for Iowa subcohort by rural status, Table S2: Positional error (m) of Version 1 and Version 2 geocodes compared to GPS by rural status.
