# Supplementary material for: Spatial Heterogeneity in Positional Errors: A Comparison of Two Residential Geocoding Efforts in the Agricultural Health Study
Source: Int J Environ Res Public Health. 2021 Feb 9;18(4):1637. doi: 10.3390/ijerph18041637 (PMC7915413; doi:10.3390/ijerph18041637)
Supplement: Supplementary file 1 [file ijerph-18-01637-s001.pdf]

Supplemental Appendix A:

Spatial Error Models:

For aspatial linear regression models that had significant spatial autocorrelation in their residuals, Simultaneous Autoregressive error models (SARerr; "spatial error model") were performed to determine the coefficients and p-values for the effects of population density and rurality on geocode improvement. We used Alkaike information criterion (AIC) to compare model fit to determine optimal k neighbors for each model ($k \in \mathbb{Z}$: $k \in [1,7]$).

The maximum likelihood estimation of a SARerr model takes the form:

$$y = X\beta + \lambda W u + \varepsilon \qquad (S1)$$

Where y is the log transformed 'improvement' value and X are the two variables (population density and urbanicity) with their coefficients β. The spatial structure λW is included in the error term u that also includes random, nonspatial error ε. λ is the spatial autoregression coefficient and W is the spatial weights matrix, here defined using k-nearest neighbor adjacency. Nearest neighbor k of 4 and 5 were chosen for Iowa GPS and Iowa Rooftop Improvement models, respectively, based on AIC model fit. The autoregression coefficient was positive (Iowa GPS: λ=0.15; Iowa Rooftop: λ=0.38) and statistically significant (Iowa GPS: $p<0.001$; Iowa Rooftop: $p<0.0001$) for both spatial regressions. The SARerr models were conducted using the spatialreg package version 1.1-5.

Spatial interpolation: Kriging

We interpolated the positional error for each study area to help identify spatial patterns. One interpolation approach is kriging, which uses a variogram model of the positional error values at point locations to predict the positional error. Spatial kriging maps (presented in Figure 1) were conducted using the gstat package version 2.0-6. The best-fitting semivariogram (smallest sum of squared error value) of the natural logarithm transformed positional error was selected for each gold standard coordinate set. The predicted distances were exponentiated to return to the linear distance.

Spatial distribution of positional error improvement value

We calculate the relative risk in the distance between a gold standard (i.e., rooftop or GPS coordinates) and their Version 1 and Version 2 geocodes to take into account the size of the geocode positional error (i.e., the distance between the gold standard and geocode; Z) at both time points. The value is a ratio and is commonly expressed logarithmically. Here, we refer to this as a relative risk "improvement." We expect this difference value to be negative, which would indicate the distance between a gold standard and the Version 2 geocodes is smaller than the distance between a gold standard and the Version 1 geocode. See an example computation below:

$$ln(Improvement_{goldstandard}) = ln\left(\frac{Z_{Version2}}{Z_{Version1}}\right) \qquad (S2)$$

where improvement is the ratio in the distances between a gold standard and the Version 1 geocode ($Z_{Version1}$) and between a gold standard and the Version 2 geocode ($Z_{Version2}$). We

restrict our comparison to participants who have a best Match Status for both Version 1 and Version 2 geocodes.

We can visualize the positional error improvement using the spatial relative risk function and presenting the logarithmic estimate (log relative risk). This function smooths the estimates from the data across our study areas. First, we visualize the spatial density of study participants within each gold standard weighted by their positional error distances. Participants with more positional error are weighted more than participants with less positional error.

The improvement in positional error can be estimated using the spatial relative risk function that takes the natural logarithm of the ratio of the Version 2 geocode density (numerator) and the Version 1 density (denominator) weighted by their respective positional error distances. The underlying density of participants is cancelled out because the same participants are used in each component of the ratio and only the positional error distances are compared. We used the spatstat package version 1.64-1 and the Jones-Diggle edge correction. In Appendix Figure 1, blue-colored zones designate areas with many participants with shorter distances between their gold standard and Version 2 geocoded address, an improvement in positional error. The red-colored zones designate areas with many participants with shorter distances between their gold standard and Version 1 geocoded addresses, a deterioration in positional error. The positional error improvement is spatially heterogeneous for all gold standards. Using the delta method, the standard error ($\hat{\sigma}_{ln(E)}$) of the log relative risk estimate ($ln(E)$) is approximately:

$$\hat{\sigma}_{ln(E)} = \frac{\hat{\sigma}_E}{E} \qquad (S3)$$

where the standard error of the relative risk estimate ($\hat{\sigma}_E$) is divided by the (non-transformed) relative risk estimate (E) at each smoothed grid location in our study area.

We determine if areas where our positional error improved or deteriorated are significantly different from an expectation of homogeneous relative risk (null value of E=1) as smoothed grid cells with a relative risk estimate (E) that exceeded a two-tailed 95% confidence interval under a normal approximation for the spatial relative risk function. Presented in Figure 1, we use a two-tailed alpha level of 0.05 and categorize any area with significant (p>0.975) improvement in positional error between Version 1 and Version 2 geocodes in blue and any area with significant (p<0.025) deterioration in positional error in red (which was not observed). Insignificantly different areas are colored grey and denote areas with no change in positional error between Version 1 and Version 2 geocodes. In a sensitivity analysis, we excluded participants (n=60) with addresses in close proximity to one another (e.g., shared or immediately adjacent residences). Omitting these participants did not change the findings.

<table>
<tr><th></th><th>Iowa Rooftop</th><th>North Carolina GPS</th><th>Iowa GPS</th></tr>
<tr><td>log Relative Risk (Version 2 error/Version 1 error)</td>
<td>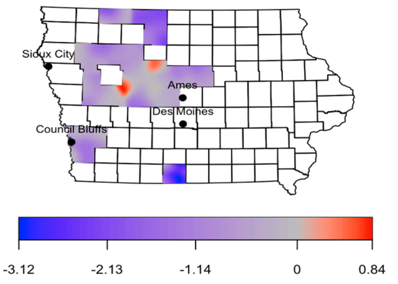
</td>
<td>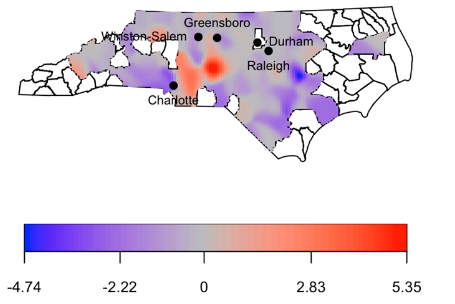
</td>
<td>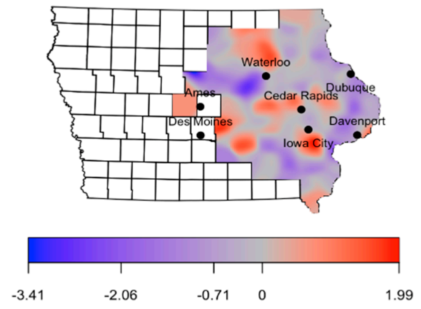
</td></tr>
<tr><td>Standard error of log relative risk</td>
<td>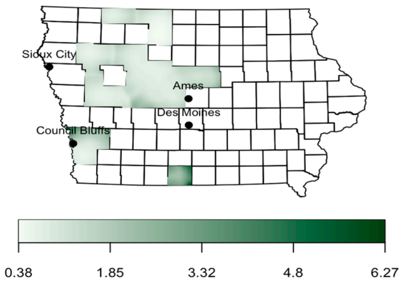
</td>
<td>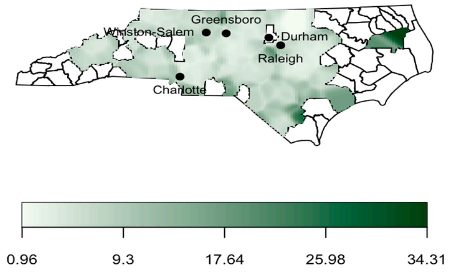
</td>
<td>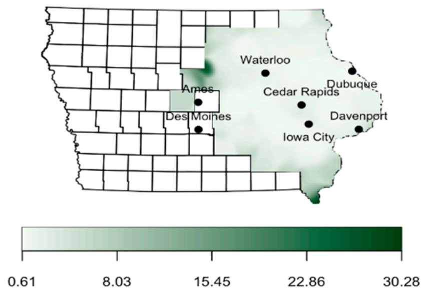
</td></tr>
</table>

Appendix Figure 1 - Spatial (log) relative risk and standard error of positional error improvement between Version 1 and Version 2 geocodes for Iowa rooftop coordinates and Iowa and North Carolina GPS coordinates.

Supplemental Tables:

Supplemental Table 1. Positional error (m) of Version 1 and Version 2 geocodes[a] compared to rooftop coordinates for Iowa subcohort by rural status

Rooftop Coordinate vs. Geocode	N	Mean (SD)	Min	5%	Positional error (m) Median (IQR)	95%	Max
**Version 1 Geocodes**							
Rural	2,827	434 (1,111)	3	37	153 (81-365)	1,267	15,172
Non-rural[b]	640	160 (739)	7	15	46 (28-69)	266	9,068
**Version 2 Geocodes**							
Rural	2,832	158 (565)	0	14	90 (42-181)	287	14,796
Non-rural[b]	608	53 (418)	1	3	13 (6-35)	80	7,779
**Improvement[c]**							
Rural	2,750	276 (1,086)	-14317	-154	54 (-15-214)	1,129	14,887
Non-Rural[b]	591	35 (111)	-535	-3	20 (0-47)	106	2,172

[a]Version 1: enrollment addresses were geocoded in 2012 for Iowa and in 2016 for North Carolina. Version 2: addresses were geocoded in 2019 for both states

[b]Non-rural location defined as the location being within a Census 2000 Incorporated Place

[c]Improvement calculated as the difference between the positional error of the Version 1 and Version 2 geocodes and limited to those with geocodes of good match status in both efforts

Supplemental Table 2. Positional error (m) of Version 1 and Version 2 geocodes[a] compared to GPS by rural status

GPS vs. Geocode		Positional error (m)						
		N	Mean (SD)	Min	5%	Median (IQR)	95%	Max
Iowa	**Version 1 Geocodes**							
	Rural	898	384 (1,057)	7	34	158 (83-345)	998	15,609
	Non-Rural[b]	70	502 (1,809)	5	10	60 (30-105)	3,382	10,407
	**Version 2 Geocodes**							
	Rural	883	254 (760)	3	21	173 (73-242)	515	15,566
	Non-Rural[b]	65	109 (419)	3	6	25 (14-59)	246	3,375
	**Improvement[c]**							
	Rural	866	134 (812)	-1,765	-305	9 (-88-144)	615	12,102
	Non-Rural[b]	65	26 (83)	-121	-88	10 (-5-45)	164	458
North Carolina	**Version 1 Geocodes**							
	Rural	251	295 (1,371)	7	27	117 (68-225)	549	19,323
	Non-Rural[b]	15	227 (367)	9	9	84 (31-171)	1,354	1,354
	**Version 2 Geocodes**							
	Rural	246	203 (1,242)	3	11	48 (25-158)	514	19,403
	Non-Rural[b]	12	33 (17)	9	9	29 (20-41)	65	65
	**Improvement[c]**							
	Rural	243	56 (207)	-1,002	-204	40 (-3-105)	332	12,102
	Non-Rural[b]	11	165 (385)	-19	-19	65 (1-121)	1,316	1,316

[a]Version 1: enrollment addresses were geocoded in 2012 for Iowa and in 2016 for North Carolina. Version 2: addresses were geocoded in 2019 for both states
[b]Non-rural location defined as the location being within a Census 2000 Incorporated Place
[c]Improvement calculated as the difference between the positional error of the Version 1 and Version 2 geocodes and limited to those with geocodes of good match status in both efforts

Supplemental Figures:

Iowa Rooftop Locations	North Carolina GPS Locations	Iowa GPS Locations
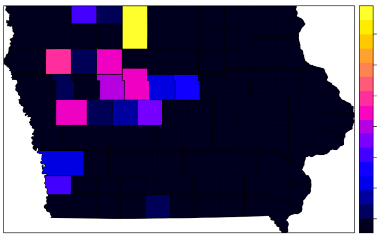	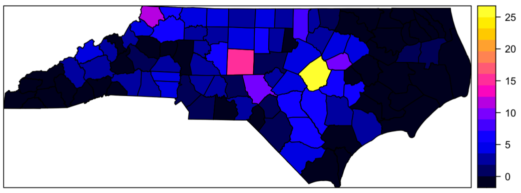	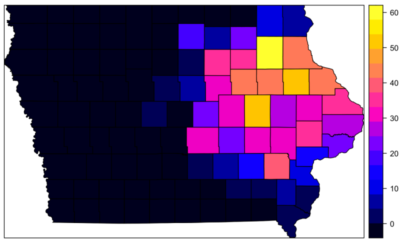

Supplemental Figure 1. Number of AHS participants per county with gold-standard rooftop and GPS coordinates in Iowa and North Carolina

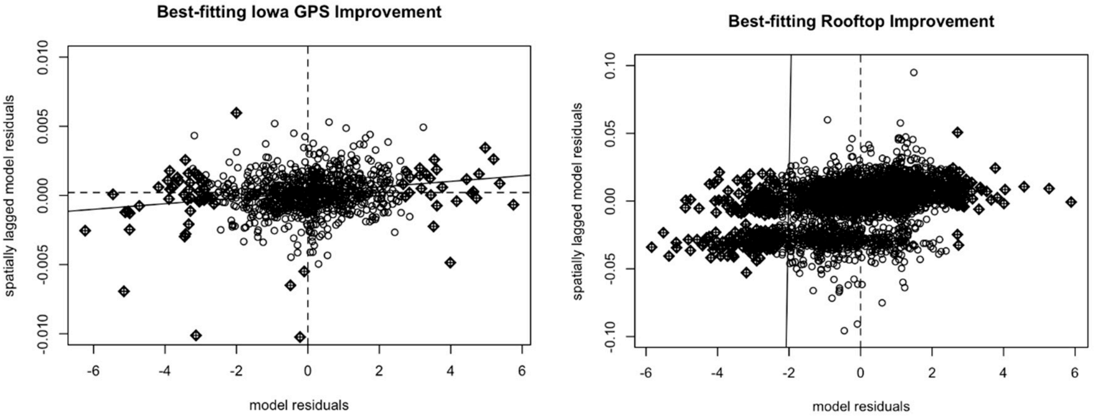

Supplemental Figure 2. Global Moran's I plots of residuals from Iowa GPS and Iowa Rooftop linear regression improvement ratio models. The following points (x,y) are not depicted in the Moran's I plots in order to rescale the y-axes; for the Iowa GPS plot: (-0.22, -0.01), (1.61, 0.03), (-3.13, -0.01), (0.16, -0.01), (0.87, 0.06); for the rooftop plot: (0.80, 1506.38), (1.43, 2659.96), (-4.94, -0.14), (0.79, 4788.47), (-5.79, -0.12), (0.81, 1479.72).
